# DNA methylation, through DNMT1, has an essential role in the development of gastrointestinal smooth muscle cells and disease

**DOI:** 10.1038/s41419-018-0495-z

**Published:** 2018-04-27

**Authors:** Brian G. Jorgensen, Robyn M. Berent, Se Eun Ha, Kazuhide Horiguchi, Kent C. Sasse, Laren S. Becker, Seungil Ro

**Affiliations:** 1 Department of Physiology and Cell Biology, University of Nevada School of Medicine, Reno, NV 89557 USA; 2 Department of Morphological and Physiological Sciences, University of Fukui, Fukui, 910-8507 Japan; 3 Sasse Surgical Associates, Reno, NV 89502 USA; 4 Gastroenterology and Hepatology, Stanford University School of Medicine, Stanford, CA 94305 USA

## Abstract

DNA methylation is a key epigenetic modification that can regulate gene expression. Genomic DNA hypomethylation is commonly found in many gastrointestinal (GI) diseases. Dysregulated gene expression in GI smooth muscle cells (GI-SMCs) can lead to motility disorders. However, the consequences of genomic DNA hypomethylation within GI-SMCs are still elusive. Utilizing a Cre-lox murine model, we have generated SMC-restricted DNA methyltransferase 1 (*Dnmt1*) knockout (KO) mice and analyzed the effects of *Dnmt1* deficiency. *Dnmt1*-KO pups are born smaller than their wild-type littermates, have shortened GI tracts, and lose peristaltic movement due to loss of the tunica muscularis in their intestine, causing massive intestinal dilation, and death around postnatal day 21. Within smooth muscle tissue, significant CpG hypomethylation occurs across the genome at promoters, introns, and exons. Additionally, there is a marked loss of differentiated SMC markers (*Srf, Myh11*, miR-133, miR-143/145), an increase in pro-apoptotic markers (*Nr4a1, Gadd45g*), loss of cellular connectivity, and an accumulation of coated vesicles within SMC. Interestingly, we observed consistent abnormal expression patterns of enzymes involved in DNA methylation between both *Dnmt1*-KO mice and diseased human GI tissue. These data demonstrate that DNA hypomethylation in embryonic SMC, via congenital *Dnmt1* deficiency, contributes to massive dysregulation of gene expression and is lethal to GI-SMC. These results suggest that *Dnmt1* has a necessary role in the embryonic, primary development process of SMC with consistent patterns being found in human GI diseased tissue.

## Introduction

The aberrant growth, or loss, of smooth muscle cells (SMCs) is a common symptom found in burdensome gastrointestinal (GI) diseases such as chronic intestinal pseudo-obstruction^1^ and megacystis-microcolon-intestinal hypoperistalsis syndrome^2^ as well as in vascular SMC (vSMC) found in atherosclerotic lesions^3^. Recent research has shown that inducing genomic DNA hypomethylation can change SMC phenotypic identity, growth patterns, and expression levels of contractile genes^4,5^, giving preliminary evidence that altering DNA methylation can shift the phenotypic identity of SMCs. Global changes in DNA methylation patterns are characteristic of digestive pathologies such as colorectal cancer^6^ and Crohn’s disease^7^.

The process of methylating DNA at cytosine residues, specifically at CpG sites, is a dynamic and reversible epigenetic modification^8^. In the mammalian genome, CpG sites can be found clustered together in regions entitled CpG islands that are often found in close proximity to, or within, transcription start sites (TSS) as well as the promoters of housekeeping and/or tissue-specific genes^9^. The addition of a methyl group to cytosine at its fifth carbon, creating 5-methylcytosine (5-mc), by DNA methyltransferases (DNMT1, DNMT3A, DNMT3B) can cause repression of associated genes as this mark, at and around the TSS, has been linked to transcriptionally repressed chromatin that denies access for transcription factors, such as serum response factor (SRF)^10,11^. Thus, as SRF is a master regulator of contractile genes in SMC^12^, the transcription of many contractile smooth muscle genes (*Myh11*, *Acta2*)^13,14^ and microRNA (miRNAs; *mir-133*, *mir-143/145*)^15,16^ is dependent on the binding of SRF to CArG (CC[A/T]_6_GG) boxes at these loci^17,18^. CArG boxes are highly conserved, have high CpG content in the region bound by SRF, are commonly found within promoter regions/close to TSS, and are recognized specifically by SRF^17^. Additionally, high CpG content sites, such as those around CArG boxes, are also well-established targets for cytosine methylation by DNA methyltransferases^19,20^. While several studies have given valuable insight into the role of DNA methylation in SMC, many of them similarly use cytidine analogs in vitro^4,5^, which are not selective in their inhibition of any specific DNMT isoform, nor do they present any efficacy of these techniques in vivo in GI-SMC.

To further this area of research, we have selectively eliminated the methyltransferase, *Dnmt1*, from the murine SMC genome. *Dnmt1* is known as the maintenance methyltransferase as its main enzymatic function is to faithfully restore methylation marks on newly formed daughter strands of DNA^21^. Deficiency of *Dnmt1* caused loss of the intestinal tunica muscularis and halted peristaltic contractions which induced intestinal dilation and death around P21. CpG methylation is significantly reduced genome-wide at promoters, exons, and introns. We also found that markers of mature SMC are lost, pro-apoptotic gene expression is increased, and *Dnmt1* knockout (KO) SMCs cytoplasmically accumulate a large number of coated vesicles. Furthermore, we noted consistent patterns of dysregulation of DNMT1 and TET3, an enzyme that initiates the demethylation of DNA^22^, between KO mice and variously diseased human GI tissue, including colon/anal cancers and Crohn’s disease. To our knowledge, this is the first in vivo evidence showing the requirement of a DNA methyltransferase, and the importance of DNA methylation, in the primary development and differentiation of GI-SMC.

## Results

### Generation, and confirmation, of deletion in SMC-restricted *Dnmt1* knockout mice

**Fig. 1:**
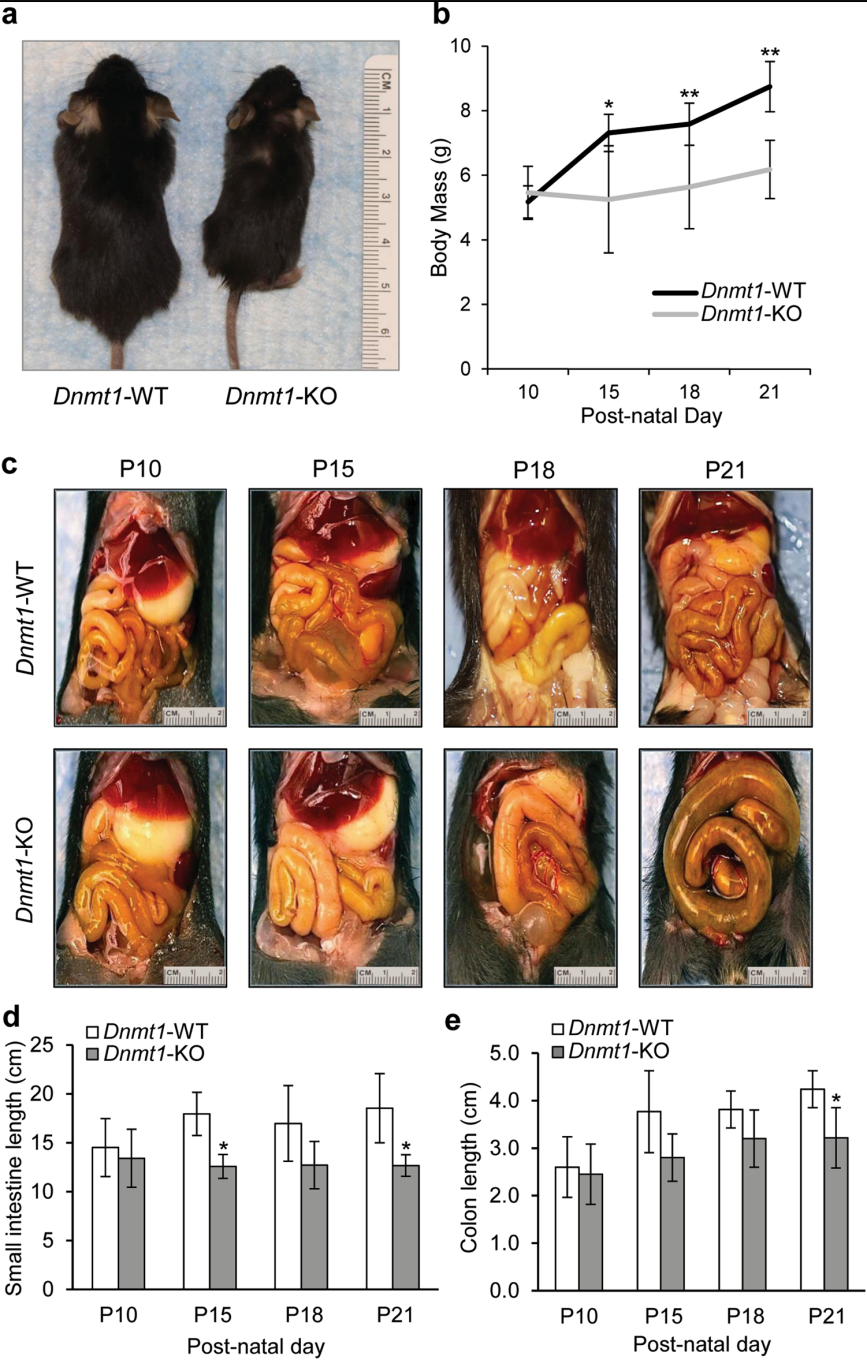
SMC-restricted congenital *Dnmt1*-KO mice are smaller in size/mass with shorter GI tracts that become increasingly dilated over time. **a** SMC-restricted congenital *Dnmt1*-KO mice at P20 are smaller when compared to *Dnmt1-*WT littermates. **b** No significant differences in body mass were seen between *Dnmt1*-KO mice and *Dnmt1-*WT mice until P15 when WT mice continued to gain mass and *Dnmt1*-KO began to lose mass. *Dnmt1*-KO slowly began to regain mass by P18 continuing into P21, most likely due to lack of peristaltic movement in the intestine causing buildup of food in the small intestine. **c** By P10, dilation can be seen in the small intestine of *Dnmt1*-KO mice with dilation increasing in size until P21 when death occurs. **d**, **e** By P21, *Dnmt1*-KO mice have significantly shortened small intestines and colons. For (**b**, **d**, **e**) *n* = 10, error bars are SD; *p < 0.005; **p < 0.001, unpaired *t*-test

As global *Dnmt1*^*-/-*^ mice are embryonic lethal^23^, it was necessary to generate a SMC-restricted *Dnmt1*^*-/-*^ KO line. By crossing *smMHC*^*Cre-eGFP/+*^, a mouse line that selectively expresses *Cre* recombinase and *eGFP* under the control of a *Myh11* promoter^24^ beginning at embryonic day 12.5, with a *Dnmt1*^*lox/lox*^ mouse line^25^, we were able to successfully generate congenital *smDnmt1*^*-/-;Cre-eGFP/+*^ (*Dnmt1*-KO) mice. In *smMHC*^*Cre-eGFP/+*^ (*Dnmt1*-WT) mice, cells that express *Cre* recombinase also express *eGFP*, thus making it possible to identify *Cre* expression through enhanced green fluorescent protein (eGFP) fluorescence. Knockout was confirmed through genotyping with primers specific for *Cre, eGFP*, known loxP sites, and deletion of floxed cassette (Supplementary Figure 1). Only those mice that were positive for *Cre*, both genomic loxP sites, and the deletion of the floxed cassette (*Dnmt1*-KO) presented the following symptoms: *Dnmt1*-KO mice were born similar in size and body mass to their wild-type (WT) littermates, but they have significantly less body mass by postnatal day 15 (P15) continuing through to P21 (Fig. 1a, b) when death occurs. Gross anatomical images show intestinal dilation beginning at P10 that continues to progress until P21 (Fig. 1c) when dilation is severe enough to cause intestinal ischemia or perforation of the deteriorated intestinal wall, causing septic shock. *Dnmt1*-KO mice also have significantly shortened GI tracts in both the small intestine (Fig. 1d) and colon (Fig. 1e) by P21.

### Dynamic changes of enzymes involved in DNA methylation in *Dnmt1*-KO mice and diseased human tissue

**Fig. 2:**
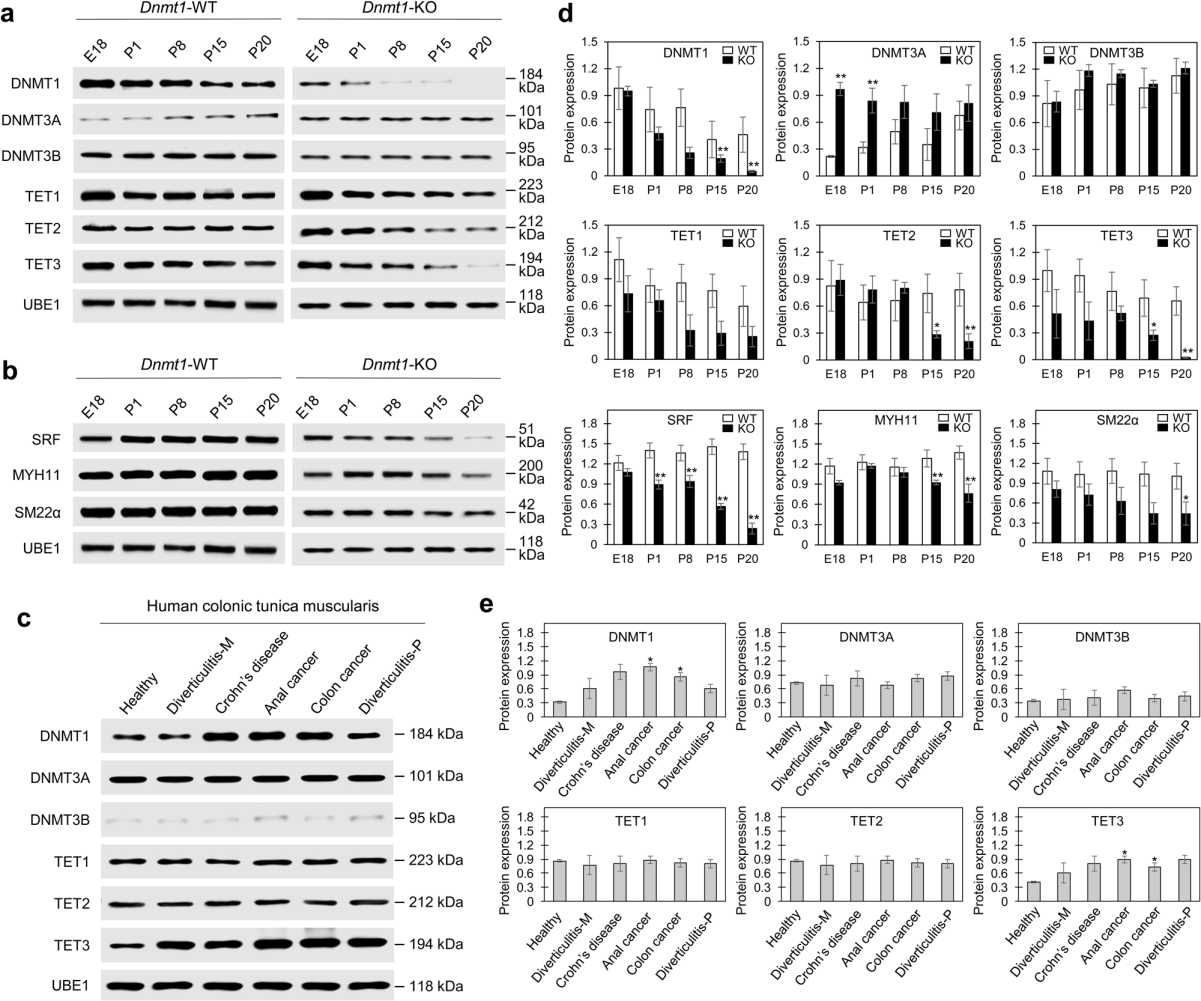
Enzymes regulating DNA methylation and mature SMC markers are dynamically altered in the jejunal tunica muscularis of *Dnmt1*-KO mice and inflamed human colonic tissue. **a** Western blotting of isolated jejunal tunica muscularis from *Dnmt1*-WT and *Dnmt1*-KO mice show temporal changes in expression of enzymes regulating DNA methylation (DNMT1, 3A and 3B) and demethylation (TET1–3). Note a progressive loss of DNMT1, and a massive reduction in expression of TET2 and TET3 in *Dnmt1*-KO mice. **b** A loss of DNMT1 correlates with significant reductions in mature SMC markers (SRF, MYH11, and SM22α) that become more pronounced with age indicating that GI-SMCs in *Dnmt1*-KO mice are indeed losing their mature, contractile status. **c** Human colonic tunica muscularis shows increased expression of DNMT1 in inflamed tissues along with TET3. Diverticulitis-M (marginal area); Diverticulitis-P (pouch). **d**, **e** Quantification of western blotting from *Dnmt1*-KO and *Dnmt1-*WT and human healthy and inflamed colonic tissue; *n* = 2–3 for mouse and human separately, error bars are SEM, *p < 0.005; **p < 0.001, unpaired *t*-test

DNA methylation is positively regulated by DNMT1/DNMT3A/DNMT3B and is negatively regulated by the Ten-Eleven Translocation (TET) family of proteins (TET1/TET2/TET3). In the tunica muscularis of *Dnmt1*-WT mice, expression levels of DNMT1 and DNMT3A are developmentally dynamic while DNMT3B is constantly expressed at all time points (Fig. 2a). DNMT1 is highly expressed at embryonic day 18 (E18) and gradually decreases from birth to P20, with the opposite pattern being seen with DNMT3A. Intriguingly, temporal expression levels of TET1/TET2/TET3 are reduced in *Dnmt1-*KO with the reductions of TET2 and TET3 being significant by P15. DNMT1 levels were drastically reduced in *Dnmt1*-KO after P1 and the protein was not detected at P20 (Fig. 2a). As previously mentioned, in *Dnmt1*-KO smooth muscle tissue, TET2 and TET3 levels were significantly reduced (Fig. 2d), suggesting that expression of these TET isoforms may be dependent on DNMT1. Furthermore, as *Dnmt1*-KO smooth muscle tissue developed, potentially compensatory DNMT3A induction occurred (Fig. 2a). Also, as DNMT1 begins to be reduced in *Dnmt1*-KO, so are markers of SMC maturity and contractibility, including SRF, MYH11, and SM22α (Fig. 2b, d). Since MYH11 and SM22α (*Tagln*) are SRF targets^12^, the reduction of SRF in *Dnmt1*-KO could result in, or exacerbate, the reduced expression of these SRF target genes and their subsequent protein products. Due to aberrant methylation patterns being identified in colorectal cancer^26^, we examined if DNMT1 is differentially expressed in the inflamed tunica muscularis from human patients with either Crohn’s disease, colon/anal cancer, or diverticulitis. We detected increases in DNMT1 as well as increases in TET3 in the inflamed smooth muscle showing that DNMT1 can regulate TET3 across species (Fig. 2c). Quantitation of protein showed that the increases in DNMT1 and TET3 were significant in the inflamed smooth muscle from anal or colon cancer samples (Fig. 2e). Intriguingly, the correlated expression of DNMT1 and TET3 in the inflamed human tissue is consistent with the smooth muscle of *Dnmt1*-WT and *Dnmt1*-KO mice (Fig. 2a), suggesting a role for DNMT1 in regulating the expression of TET3.

### *Dnmt1*-KO mice gradually lose tunica muscularis and necessary contractile proteins and accumulate coated vesicles in SMCs

**Fig. 3:**
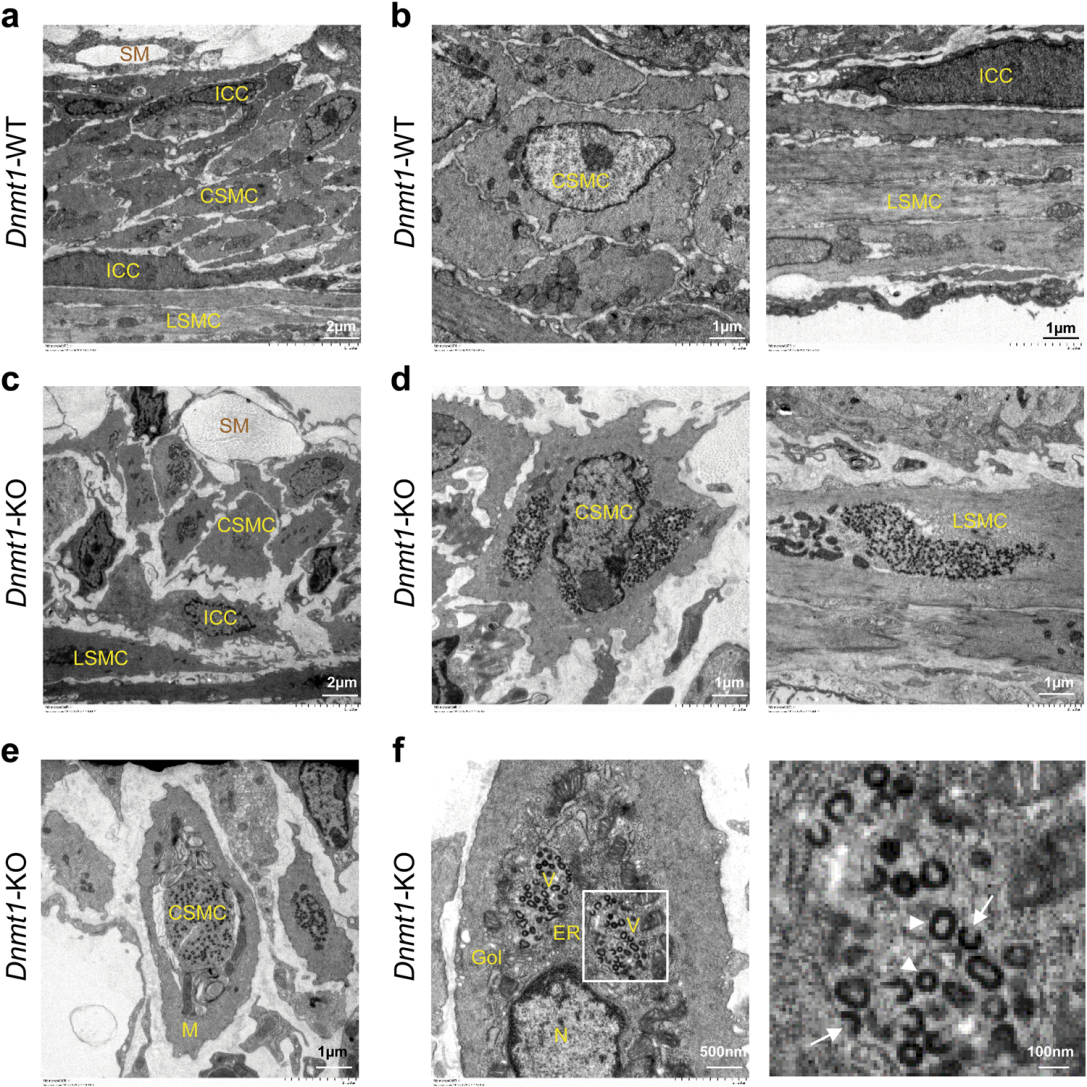
Electron microscopy (EM) reveals degeneration of smooth muscle and presence of coated vesicles in *Dnmt1*-KO SMC. **a, b** EM cross-section images of P21 *Dnmt1-*WT jejunum. **c–f** EM cross-section images of P21 *Dnmt1-*KO jejunum. Both the circular (CM) and longitudinal muscle (LM) layers in *Dnmt1-*WT jejunum are intact (**a, b**), complete with organized nuclei in SMC as well as the presence of ICC (ICC), and submucosal cells (SM). Degenerated SMC within the tunica muscularis in the jejunum of *Dnmt1*-KO mice (**c**,** d**) show cellular fragmentation, as well as a loss of nuclear organization. **e** A portion of a degenerated SMC with coated vesicles from a *Dnmt1*-KO mouse found phagocytosed in a macrophage (M). **f** Degenerated SMCs in *Dnmt1*-KO mice accumulate coated vesicles (V) trapped in the vicinity of the endoplasmic reticulum (ER) and Golgi (Gol), open vesicles: arrows; closed vesicles: arrowheads

Hematoxylin and eosin staining revealed that *Dnmt1-*KO mice begin to lose both muscle layers of the tunica muscularis at noticeable levels by P15 but did not have a significant loss of either layer until P21, with the more drastic tissue loss being found in the circular layer (Supplementary Figure 2). To confirm that the loss of tissue was indeed due to a loss of mature SMCs, immunohistochemical analysis was performed. As MYH11 is the most selective marker of mature and contractile SMCs^27^, antibodies targeting MYH11 were used to confirm the loss of mature SMCs alongside the endogenous eGFP reporter expression based on *Myh11* expression. As expected, MYH11 levels in the tunica muscularis of *Dnmt1-*WT mice continued to increase from P10 to P21, while in *Dnmt1-*KO MYH11 levels waned, starting at P15 until there was no detectable MYH11 signal at P21, indicating a complete loss of mature SMCs (Supplementary Figure 2D). The loss of MYH11 in the SMCs of *Dnmt1-*KO mice results in SMCs that are no longer contractile, leading to a weakened tunica muscularis, allowing for intestinal distention. Electron microscopy of the tunica muscularis from P21 *Dnmt1-*KO mice revealed a loss of intercellular connections between SMCs, submucosa cells, and interstitial cells of Cajal (ICC), the necessary pacemaking cells in peristaltic contractions^28^ (Fig. 3c). We also noted cellular fragmentation and the loss of nuclear/nucleolar organization within *Dnmt1-*KO SMCs (Fig. 3c, d). Furthermore, we were able to detect macrophages engulfing the deteriorated SMCs in *Dnmt1-*KO mice (Fig. 3e). Finally, we found that almost all *Dnmt1-*KO SMCs contained small coated vesicles (Fig. 3d, f), which were not associated with *Dnmt1-*WT SMC (Fig. 3a, b). *Dnmt1-*KO SMC appeared to accrue coated vesicles in their cytosol that congregated in distinct regions around the endoplasmic reticulum and Golgi (Fig. 3f).

### Transcriptomic analysis of tissue/SMC reveals loss of mature SMC markers and miRNAs and increases in lipid, immune processing, and pro-apoptotic genes

**Fig. 4:**
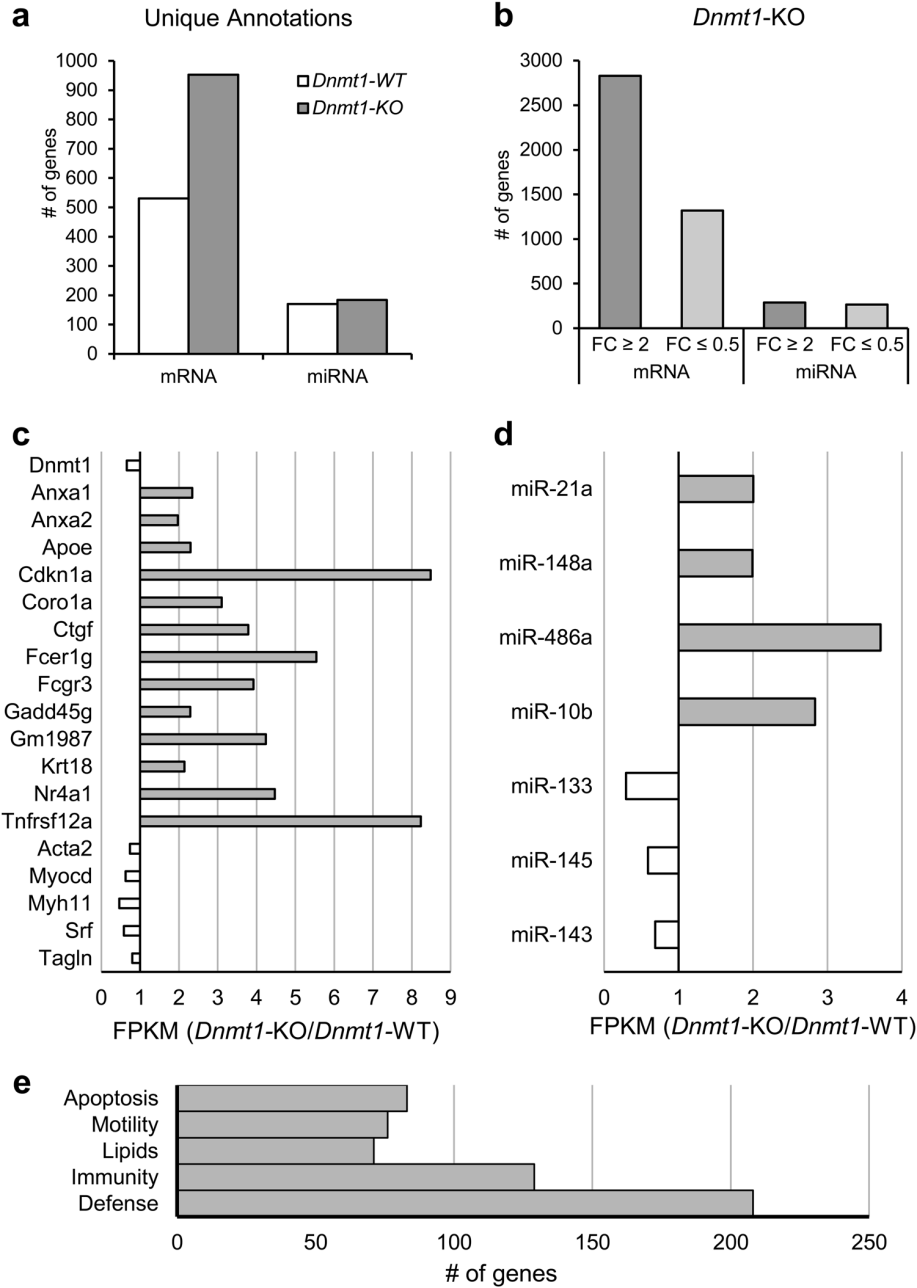
Transcriptomic analysis reveals dramatic changes of expression in *Dnmt1*-KO jejunal tunica muscularis. **a**,** b** The expression profile of *Dnmt1*-KO jejunal tunica muscularis shows a higher number of unique mRNA transcripts and more genes that increased their expression than reduced their expression when compared to *Dnmt1*-WT. Changes in overall miRNA expression were not as dramatic. **c** Increases in several genes associated with apoptosis or lipid processing were upregulated in *Dnmt1*-KO mice. Additionally, transcript levels of SMC markers *(Srf, Myh11*) are reduced along with the SRF transcriptional co-activator*, Myocd*. **d** Although overall miRNA changes are not as pronounced as mRNA changes in *Dnmt1*-KO mice, there are select miRNAs that increase their expression level. **e** A gene ontology evaluation uncovered the prevalence of five general categories enriched in *Dnmt1*-KO jejunal tunica muscularis with many genes relating to defense, immunity, lipids, motility, and apoptosis being found

To gather a more complete understanding of the gene expression changes happening in *Dnmt1*-KO mice, we performed a transcriptomic analysis on the jejunal tunica muscularis of both *Dnmt1*-KO and *Dnmt1*-WT mice. Summaries and full expression profiles of messenger RNA (mRNA)/miRNA sequencing results can be found in Supplementary Table 1 and Supplementary Table 3, respectively. The transcriptome of *Dnmt1*-KO has 954 unique mRNA gene annotations, compared to only 530 in *Dnmt1*-WT with approximately equal amounts of unique miRNA annotations between *Dnmt1*-KO (184) and -WT (170) mice (Fig. 4a). When compared to *Dnmt1*-WT mice, *Dnmt1*-KO mice have 2,829 genes that at least double their expression and only 1,319 that lose at least half their expression, with similar amounts of both upregulated (289) and downregulated (265) miRNA transcripts (Fig. 4b). The differences between the relative amount and type of mRNA/miRNA transcripts expressed exposes a remodeled transcriptome in *Dnmt1*-KO mice. *Dnmt1*-KO mice have considerable losses of SMC marker transcripts necessary for mature functioning, including *Acta2* (25.7% loss), *Myocd* (37.4% loss), *Myh11* (53.2% loss), *Srf*(41.7% loss), and *Tagln* (19.9% loss) (Fig. 4c) as well as reductions in necessary SMC miRNAs miR-143 (31.1% loss), miR-145 (40.8% loss), and miR-133 (70.4% loss) (Fig. 4d). Since the transcription factors SRF and MYOCD are required for *Myh11* expression^29^, and the previously mentioned miRNAs^30^, the reduction of *Srf* and *Myocd* exacerbates the deterioration of SMCs as *Srf* expression in Dnmt1-KO does not become significantly reduced until P15 (Supplementary Figure 3) and therefore is likely not the primary insult involved in the loss of GI-SMCs. We also noted increases in miRNAs associated with regulating cellular identity and proliferation including, miR-10b^31^, miR-21a^32^, miR-486a^33^, and miR-148a. MiR-148a is an established inhibitor of *Dnmt1* expression^34^, thus *Dnmt1*-KO SMCs, through increased miR-148a, likely create even further repression of *Dnmt1* expression. Furthermore, both miR-10b and miR-148a expression are known to regulated by genomic CpG methylation^31,35^. For mRNA analysis, we selected 392 overexpressed genes in the tunica muscularis of *Dnmt1*-KO mice and 365 of them had some level of significant demethylation (Supplementary Figure 4). Gene ontology (GO) examination revealed that the most overrepresented biological process categories included those associated with lipids, apoptosis, defense responses to stress/stimuli, and immune responses (Fig. 4e and Supplementary Table 4). The quantitative increase in expression levels of many of these genes that were found to be upregulated in *Dnmt1*-KO varied widely (Fig. 4c).

**Fig. 5:**
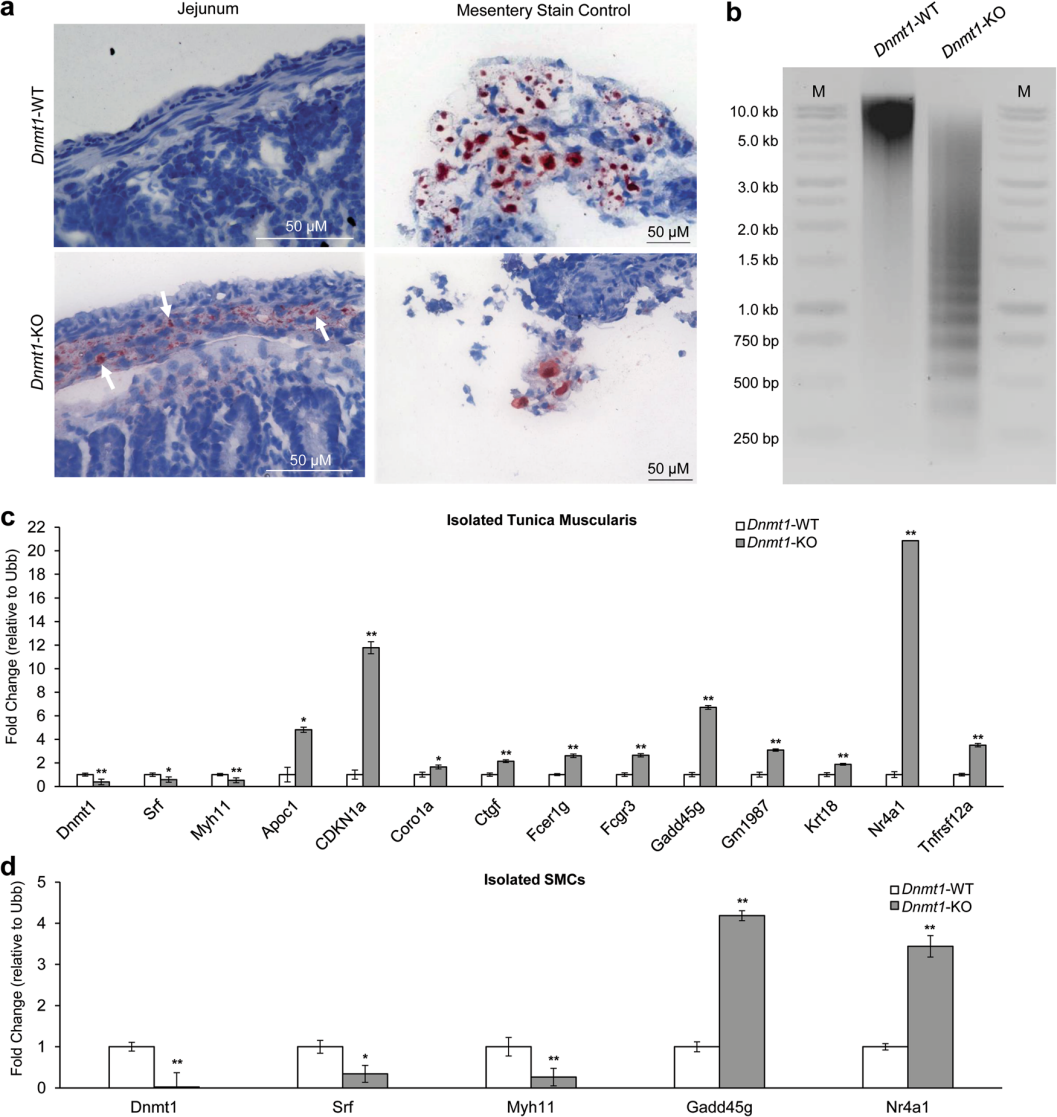
*Dnmt1*-KO tunica muscularis accumulates lipids, has increased expression in apoptotic genes and characteristic apoptotic DNA fragmentation. **a** Oil Red O staining of jejunal cross-sections reveals an accumulation of lipids in the muscle layer of *Dnmt1*-KO mice (white arrows; red stain) that is not seen in *Dnmt1*-WT mice. **b** At P21, a clear fragmentation of extracted DNA can be seen in the isolated tunica muscularis of *Dnmt1*-KO mice. Distinct fragmentation patterns of genomic DNA are generated due to cleavage at specific sites, a characteristic of apoptotic cells. **c** qPCR of isolated tunica muscularis at P15. As expected, *Dnmt1, Srf*, and *Myh11* all have significantly reduced expression in *Dnmt1*-KO tunica muscularis as well as significant increases in many genes linked with lipid processes and/or pro-apoptotic pathways (*n* = 4, error bars are SEM, *p < 0.05, **p < 0.01, unpaired *t*-test). **d** In isolated *Dnmt1*-KO SMC at P15, more dramatic losses of *Dnmt1, Srf*, and *Myh11* were observed as well as increases in the pro-apoptotic markers *Gadd45g* & *Nr4a1* (*n* = 2, error bars are SEM, *p < 0.05, **p < 0.01, unpaired *t*-test)*. Ubb* was used as an endogenous control in (**c**, **d**)

We selected upregulated gene groups associated with lipids and apoptosis for further study. Oil red O staining of cross-sections from *Dnmt1-*KO mice confirmed an accumulation of lipids in their jejunal tunica muscularis (Fig. 5a), consistent with our mRNA-sequencing and GO term analysis. Next, we examined if *Dnmt1-*KO SMCs were undergoing apoptosis. DNA fragmentation is a hallmark of apoptosis^36^ and was observed in *Dnmt1-*KO tunica muscularis at P21 (Fig. 5b). Our transcriptome represents the entirety of the tunica muscularis where the main population of cells are SMCs. However, immune cell infiltration into the tunica muscularis may be responsible for the increases in genes associated with immunity and other GO categories. With the use of flow cytometry, we removed cells from the tunica muscularis that were CD45^+^, a known marker of immune cells^37^, then selected for cells that were CD45^−^ and eGFP^+^ in P15 *Dnmt1-*KO and *Dnmt1-*WT. Indeed, the results of our flow cytometry identified ~5-fold increase in CD45^+^ cells within *Dnmt1-*KO tissue when compared to *Dnmt1-*WT mice while CD45^-^ and eGFP^+^ SMC were reduced ~3-fold (Supplementary Figure 5). Sorted CD45^-^, eGFP^+^ cells represented isolated SMCs. We confirmed that isolated SMCs from *Dnmt1-*KO mice had almost complete ablation of *Dnmt1*, significant reductions in *Myh11* and *Srf* expression, and dramatic increases in the pro-apoptotic genes *Gadd45g* and *Nr4a1* (Fig. 5d). However, we found other genes related to apoptosis and lipids upregulated in P15 tissue that were not upregulated in isolated *Dnmt1-*KO SMCs, indicating that their upregulation is likely due to immune cells infiltrating into the tunica muscularis (Fig. 5c, d).

### Loss of *Dnmt1* induces global genomic CpG hypomethylation

**Fig. 6:**
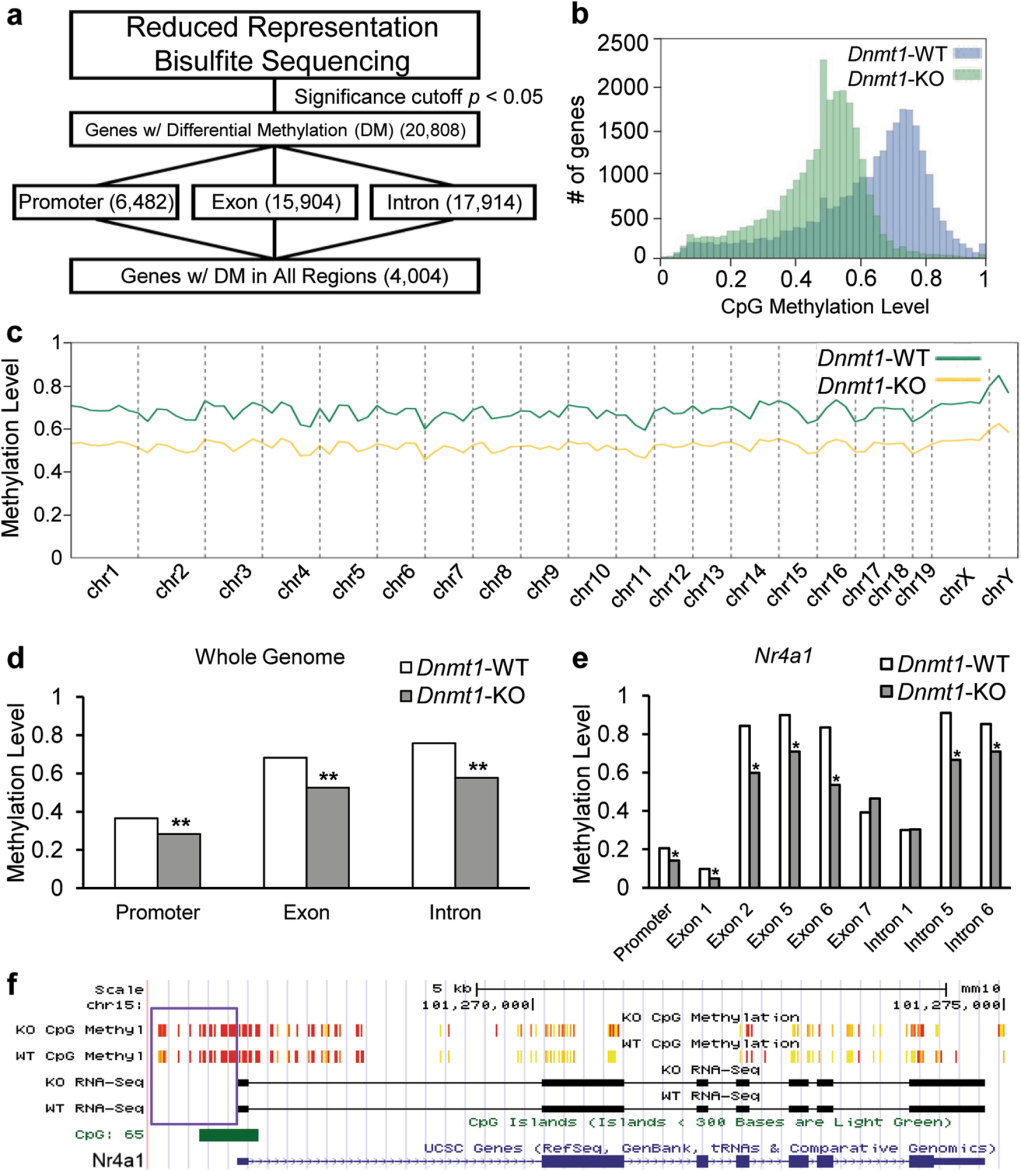
Loss of *Dnmt1* in GI-SMC causes global genomic CpG hypomethylation. **a** Reduced representation bisulfite sequencing of *Dnmt1*-KO mice compared to *Dnmt1*-WT mice demonstrates that most genes in *Dnmt1-*KO have at least one CpG site with significant levels of hypomethylation. **b** CpG methylation is reduced ~20% in *Dnmt1*-KO mice when compared to *Dnmt1*-WT mice. **c** While there is an ~20% reduction of CpG methylation in *Dnmt1*-KO mice, the patterns of methylation across each chromosome is not altered. **d** Globally, across the genome, CpG methylation is significantly reduced at promoters, exons and introns (**p < 0.00001, Fisher’s exact test). **e** Pro-apoptotic marker *Nr4a1* shows significant losses of methylation at various genomic locations, including the promoter and exon 1 (*p < 0.01, Fisher’s exact test). **f** A UCSC genome browser view of the *Nr4a1* gene with methylation tracks added (yellow=more methylated, red=less methylated) and the promoter region boxed in purple

**Fig. 7:**
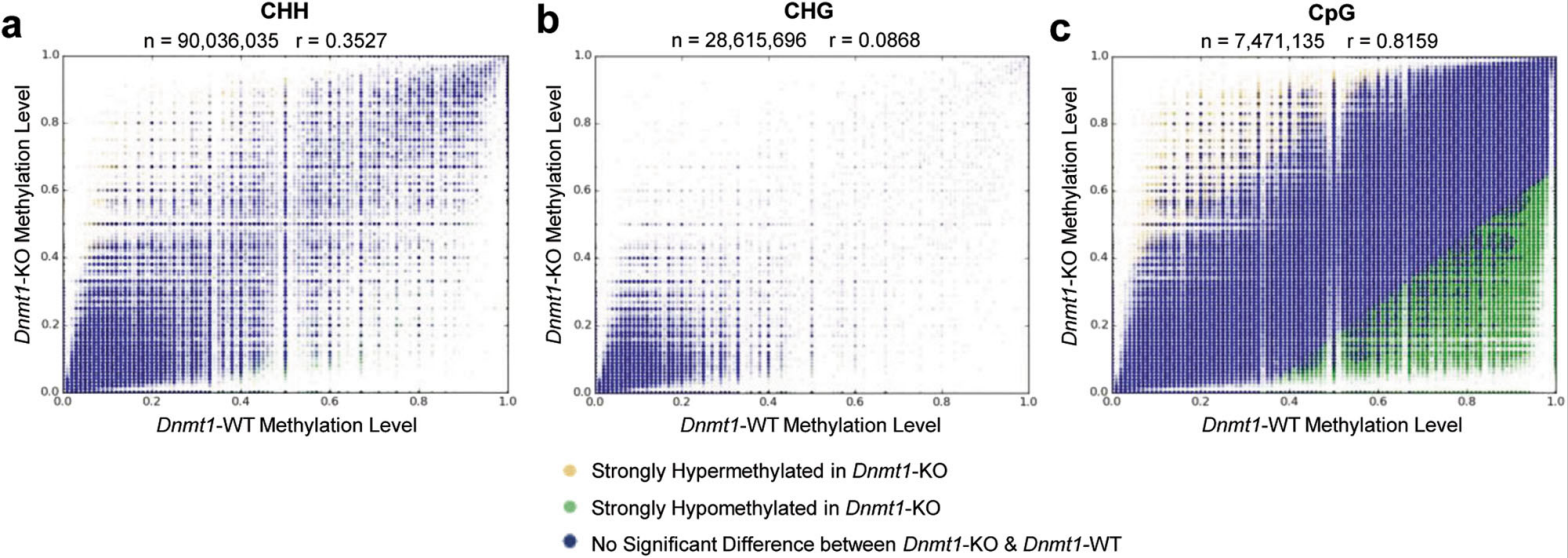
Only CpG sites show large loss of methylation in *Dnmt1*-KO. **a**,** b** While CHH sites and CHG sites did have individual sites that changed their methylation status in *Dnmt1*-KO mice, there was no strong genome-wide change in methylation status. **c** At CpG sites in *Dnmt1*-KO mice, there was marked hypomethylation at multiple site across the genome which was not observed in either CHH or CHG sites. Each dot represents an individual cytosine that mapped to both *Dnmt1*-KO and *Dnmt1*-WT samples; *r* is Pearson’s correlation coefficient and *n* is the total number of overlapping sites for each type of cytosine site

Employing reduced representation bisulfite conversion sequencing, we discovered that ~90% (20,808) of annotated genes in *Dnmt1-*KO mice had at least one CpG site with significant demethylation in either their promoter (−1 kb of TSS), exons, or introns and ~17% (4,004) of genes had at least one CpG site with significant demethylation in all sites, encompassing their promoter, exons, and introns (Fig. 6a) with genomic hypomethylation being significant, on a genome-wide scale, across annotated promoter, exon, and intron CpG sites (Fig. 6d). None of these patterns were observed for genomic CHG or CHH (H can be A, C, or T) sites (Fig. 7a, b). Across the genome of *Dnmt1-*KO mice, there was a ~20% loss of CpG methylation (Fig. 6c) with a majority of genes reducing their average CpG methylation levels from 0.8–0.6 to 0.6–0.4 (Fig. 6b). We also observed CpG sites that were strongly hypermethylated in *Dnmt1*-KO mice, but these sites were considerably outnumbered by the amount of strongly hypomethylated CpG sites (Fig. 7c). We created browser tracks that visualize and quantify the changes in CpG methylation within the University of California, Santa Cruz (UCSC) genome browser^38^ for any annotated genomic site. In addition, we added transcriptome level RNA-sequencing (RNA-seq) data for both mRNA and miRNA from jejunal smooth muscle in both *Dnmt1-*KO and *Dnmt1-*WT mice (Supplementary Table 3) to the browser. The browser tracks were installed at our Smooth Muscle Transcriptome Project webpage: https://med.unr.edu/physio/transcriptome, entitled “UCSC Smooth Muscle Methylome Browser.” This browser provides a comprehensive reference for transcriptomic and genomic DNA methylation status at CpG sites in the jejunal smooth muscle of *Dnmt1*-WT and *Dnmt1*-KO mice. The browser can also interact with genome-level bioinformatics (e.g., ENCODE) data publically available in the UCSC Genome Browser. In *Dnmt1-*KO mice, *Nr4a1* has significant losses of methylation at its promoter as well as exons 1, 2, 5, and 6 and introns 5 and 6 (Fig. 6e), as shown on the browser (Fig. 6f), suggesting the expression of the pro-apoptotic *Nr4a1* could be regulated by CpG methylation levels controlled by DNMT1, agreeing with previous findings^39^. The entirety of our CpG methylomic results and analysis for every annotated promoter, exon, and intron can be found in Supplementary Table 5.

## Discussion

By eliminating *Dnmt1* from the murine genome in a SMC-restricted model, we showed the in vivo necessity of *Dnmt1* in the embryonic development of GI-SMCs. SMC-restricted congenital *Dnmt1-*KO mice have shortened GI tracts, and lose their tunica muscularis by P21. *Dnmt1-*KO GI-SMC have significant genomic CpG demethylation, a loss of intercellular connectivity, decreases in necessary SMC transcripts (*Myh11, Srf*, miR-133, miR-143/145), and increases in both pro-apoptotic genes (*Gadd45g, Nr4a1*) and miRNAs associated with changes in cellular identity (miR-21a, -148a, -186a, -10b). In fact, we found aberrant hypermethylation flanking a CArG box (CCATATAAGG: antisense) and SRF binding site found within the second intron of *Srf* in *Dnmt1*-KO (Supplementary Figure 6) that may have contributed to the reduction *Srf* expression, thus increasing the apoptotic potential of *Dnmt1*-KO SMC. Our results are similar to both congenital and inducible intestinal epithelial cell-specific *Dnmt1*-KO models. Both models show genomic hypomethylation, DNA damage, and increased apoptosis in affected *Dnmt1* knockout cells^40,41^. In the congenital intestinal epithelial model, only 35% of *Dnmt1* knockout mice live until P20, similar to our SMC-restricted *Dnmt1*-KO mice which die around P21 and none have ever survived past P24. When *Dnmt1* is inducibly ablated from intestinal epithelial cells at later stages of development, the mouse does not die and sees an increase in *Dnmt3b* expression, which also directly compensated for the loss of DNA methylation due to the knockout of *Dnmt1*. This pattern of compensatory DNMT isoform induction due to loss of a separate DNMT could potentially be why we see increased DNMT3A seen during development in our *Dnmt1*-KO mice, as DNMT3A has been shown to be able to maintain methylation patterns in mouse embryonic stem cells lacking DNMT1^42^.

While it is clear that GI-SMCs in congenital *Dnmt1-*KO mice are undergoing remodeling and apoptosis, it is not clear what the causative insult of these changes is, and it is more likely a combination of several pro-apoptotic influences including genomic CpG hypomethylation, epigenome modulation, lack of cellular connectivity, upregulation of pro-apoptotic genes/miRNAs, downregulation of required SMC proteins/miRNAs, exposure to lipoproteins, and chromosomal instability. As it pertains to pro-apoptotic genes upregulated in *Dnmt1*-KO SMCs, *Gadd45g* is a marker of DNA damage, activator of the p38/c-Jun N-terminal kinase apoptotic pathway^43^ and has been correlated with increased expression in a double knockout of *DNMT1* and *DNMT3B *in vitro^44^. *Nr4a1* is an orphan nuclear receptor that has been shown to be regulated by DNMT1 and the methylation level of its promoter region^39^. Expression of *Nr4a1* is associated with an induction of apoptosis through multiple pathways^45^, as well as direct inhibition of the growth of SMCs^46^. As for genomic CpG hypomethylation, it is likely a causative feature involved in the transcriptomic remodeling of mRNA and miRNA expression, but probably does not account for all changes. Only about 10% of all miRNAs have been shown to change expression based on levels of DNA methylation^47^, denoting a secondary role of DNMT1 in regulating miRNA expression in GI-SMCs. DNMT1 interacts with up to 58 transcription factors^48^, and many epigenetic modifiers^49^, and thus it is probable that loss of *Dnmt1* has downstream deleterious consequences outside of genomic hypomethylation. Furthermore, in *Dnmt1*-null cells, DNA mismatch repair is impeded and microsatellite instability increases ~4-fold^50^, indicating a pivotal, methylation-independent role of DNMT1 in DNA damage repair. While previous in vitro studies of complete *Dnmt1* knockout in mouse embryonic stem cells have reported more dramatic genomic CpG methylation reductions from ~75% to ~18%^51^ or lower at specific sites^52^, cell-selective in vivo knockout of *Dnmt1* in intestinal epithelial results in CpG methylation reductions from ~75% to ~45%^40,41^, similar to our own in vivo findings (Fig. 6c).

Additionally, the observed lack of nuclear/nucleolar organization in electron microscopy images of *Dnmt1*-KO could be indicative of catastrophic chromosomal instability. Of the 25 genes in the CIN25 gene signature of chromosomal instability^53^, 23 were found to be upregulated in *Dnmt1*-KO smooth muscle tissue (data not shown). We were also able to show that congenital *Dnmt1-*KO mice have reduced levels of all TET proteins, and thus it is likely that *Dnmt1-*KO mice cannot properly regulate 5-hmc or 5-mc levels, causing dysregulation of the entire DNA methylation apparatus. We also showed that expression levels of DNMT1 and TET3 are associated and correlated within the inflamed tunica muscularis of colorectal cancer tissue as well as in the jejunal tunica muscularis of both *Dnmt1*-WT and *Dnmt1*-KO mice. Further studies are needed to elucidate the associations and interactions between DNMT1 and TET3 in inflamed and injured smooth muscle.

Most surprisingly, *Dnmt1-*KO SMC show an accumulation of lipids and lipid associated transcripts within their tunica muscularis. Many of the genes upregulated in our transcriptomic analysis are commonly associated with vSMC containing atherosclerotic plaques^54^. In vSMC, lipoproteins, based on oxidation status, have been implicated in changing the phenotypic identity of SMCs into either a proliferative or apoptotic fate^55^.

In conclusion, the lack of *Dnmt1* in GI-SMC resulted in an inability to maintain de novo methylation patterns and the consequences of this loss become exacerbated with each round of mitotic division. As can be surmised, further research into all observed phenotypic aspects of *Dnmt1*-KO is necessary to understand all cellular consequences of DNMT1 deficiency.

## Methods

### Mice

In a C57BL/6 background, smMHC^Cre-eGFP/+^ mice^24^ were crossed with Dnmt1^loxllox^ mice^25^ to make smDnmt1^-/-;Cre-eGFP/+^ mice. PCR confirmed genotypes (Supplementary Figure 1). Primer sequences are found in Supplementary Table 2. Mice were bred, maintained, and euthanized following guidelines set by the Institutional Animal Care and Use Committee at the University of Nevada-Reno Animal Resources.

### Human tissue

Isolated human GI smooth muscle tissue sections were donated by Dr. Kent Sasse (Sasse Surgical Associates) and Dr. Laren S. Becker (Stanford University School of Medicine). Use of human tissue was approved by the Institutional Review Board at the University of Nevada, Reno.

### Tissue isolation and preparation

The GI tract was extracted into 1× Hank’s Calcium Free Buffer. Tissue then underwent one of two processes: (1) smooth muscle isolation and (2) whole tissue preparation for cross-sectioning. (1) The muscle layer was peeled from the mucosa for use in extractions/experiments. (2) The tissue was fixed in 4% paraformaldehyde (PFA), a dehydration in 20% sucrose/1× phosphate-buffered saline (PBS), then placed in 1:1 OCT/20% sucrose and super-cooled by liquid nitrogen. Using a cryostat microtome (Leica, Wetzlar, Germany), 8 µM thick tissue sections were cut onto slides coated with VectaBond (Maravai Biosciences, San Diego, CA, USA), and allowed to dry. Prepared slides were used for downstream immunohistochemical/histological staining.

### Isolation of total and small RNAs

Isolated muscle tissue was placed in lysis buffer and homogenized by bead beating in an air-cooled Bullet Blender Storm (Next Advance, Troy, NY, USA). Total RNAs and miRNAs were isolated from smooth muscle and sorted SMCs using mirVana miRNA Isolation Kit (Ambion, Foster City, CA, USA) as previously described^56^. Extracted total RNAs and small RNAs were used for quantitative PCR (qPCR) and RNA-seq.

### Quantitative PCR

Extracted total RNA was reverse transcribed into complementary DNA (cDNA) using SuperScript III Reverse Transcriptase (Invitrogen, Carlsbad, CA, USA), after DNA removal with DNA*-*free DNA Removal Kit (Ambion). cDNA samples were then quantified, diluted, and tested for quality. Standard qPCR protocol was carried out on a 7900HT Fast Real-Time PCR System (Applied Biosystems, Foster City, CA, USA) as previously described^56^. Primer sequences are found in Supplementary Table 2.

### Immunohistochemistry

Slides were exposed to 1× Tris-buffered saline (TBS)/0.1% Tween 20 followed by exposure to 4% milk/1× TBS/0.1% Tween 20. Slides were then incubated at 4 °C with 1° antibody. The following day, slides were incubated with 2° antibody at room temperature. After washing the slides in 1× TBS, the slides were mounted and allowed to cure. See Supplementary Table 2 for antibodies/dilutions. Slides were imaged with an Olympus FluoView FV1000 (Tokyo, Japan) confocal microscope.

### Hematoxylin and eosin staining

Cross-section slides were exposed to hematoxylin, rinsed with water, and then stained in eosin Y. After several dehydrating baths of increasing ethanol (EtOH) concentration (80%, 95%, 100%) and two terminal xylene baths, the slides were mounted and allowed to cure. Slides were imaged with an Olympus BX43 (Tokyo, Japan) brightfield microscope.

### Oil Red O staining

Cross-section slides were exposed to pure propylene glycol, then moved to 0.5% Oil Red O solution at 60 °C followed by soaking in 85% propylene glycol. slides were then rinsed and counterstained with hematoxylin, rinsed with water, then mounted with aqueous mounting reagent and allowed to cure. Slides were imaged with a Keyence BZ-x710 (Osaka, Japan) brightfield microscope.

### Electron microscopy

Whole jejunum and colon tissue were fixed (3% glutaraldehyde and 4% PFA in 0.1 M phosphate buffer) for several days at room temperature. Specimens were then fixed in 1% OsO_4_ at 4 °C, rinsed in distilled water, then block stained with saturated aqueous uranyl acetate solution, followed by dehydration in a graded series of EtOH and finally embedded in EPON 812. Ultrathin sections were stained with uranyl acetate and lead citrate and then examined using a Hitachi H-7650 (Tokyo, Japan) microscope.

### Western blot

Isolated SM tissue was ground by mortar and pestle in modified RIPA buffer. Transferred polyvinylidene fluoride membranes were pre-blocked in 5% milk, then exposed to 1° antibody at 4 °C in 2% milk/0.05% Tween 20 at varying dilutions. The membranes were exposed to 2° antibody at room temperature. All antibodies used can be found in Supplementary Table 2. Imaging by UVP EC3 (Upland, CA, USA) Imaging system. Quantification of western blot banding patterns was done using ImageJ and histograms were produced in SigmaPlot (Systat, San Jose, CA, USA).

### DNA methylome

Genomic DNA was isolated from jejunal tunica muscularis at P15 (2 males and 2 females for both *Dnmt1-*KO and *Dnmt1*-WT; *n* = 4) using Qiagen (Hilden, Germany) AllPrep DNA spin columns. DNA samples were pooled and shipped to Zymo Research Corporation (Irvine, CA, USA) where the samples underwent Methyl-MidiSeq which involves genomic DNA extraction, fragmentation, end modification, bisulfite conversion, limited amplification, and sequencing. After sequencing, bioinformatic processing was utilized in comparing amount of methylation at CpG, CHH, and CHG sites between *Dnmt1-*WT and *Dnmt1-*KO samples. Methylation ratios were calculated (methylated cytosines/total cytosine reads) for individual sites and then averaged across regions (exons, introns, promoters = −1 kb of TSS). These averages were then used in Fisher’s exact test to quantify significant changes (*p* < 0.05) in methylation. The reference genome used was GRCm38/mm10. The data from our bisulfite conversion sequencing were submitted to the Gene Expression Omnibus (GEO) repository with the following accession numbers: GSM2947783, *Dnmt1*-WT jejunum; GSM2947784, *Dnmt1*-KO jejunum.

### RNA sequencing

Total RNAs and small RNAs isolated from *Dnmt1-*KO and *Dnmt1-*WT jejunal tunica muscularis at P15 (2 males and 2 females for both *Dnmt1-*KO and *Dnmt1*-WT; *n* = 4) were shipped to LC Sciences (Houston, TX, USA) where mRNA-seq and miRNA-seq were performed. Sequencing data analysis, alignments, annotations, and bioinformatics processing were carried out as previously described^57^. The reference genome used was GRCm38/mm10. Our RNA-seq data for both mRNA and miRNA were submitted to the GEO repository with the following accession numbers: GSM2936348, *Dnmt1*-WT jejunum mRNA; GSM2936349, *Dnmt1*-KO jejunum mRNA; GSM2936350, *Dnmt1*-WT jejunum miRNA; GSM2936351 *Dnmt1*-KO jejunum miRNA.

### Cell sorting

Cells were dispersed as previously described^58^. Cells were incubated with APC-Cy7 Anti-CD45 (Biolegend, Clone 30-F11, 1.0 μg/mL; San Diego, CA, USA) followed by washing with PBS/1% FBS. Resuspended cells had Hoechst 33258 (1 μg/mL) added as a viability marker. Cells were sorted and analyzed using the BD Biosciences (San Jose, CA, USA) FACSAria II Special Order Research Product with a 130 μm nozzle with sheath pressure at 12 psi. The 355 nm laser excited Hoechst 33258 with a 450/50 nm bandpass filter. The eGFP was excited using a 488 nm laser with a 530/30 nm bandpass filter. A neutral density filter 2 was used on the forward scatter detector due to the high forward scatter properties. Cells that were CD45^-^ and eGFP^+^ were sorted into PBS/1% FBS. Acquisition was performed on BD FACSDiva 8.0 and TreeStar Flowjo (Ashland, OR, USA) was used to generate figures.

## Electronic supplementary material

Supplementary Fig. 1

Supplementary Fig. 2

Supplementary Fig. 3

Supplementary Fig. 4

Supplementary Fig. 5

Supplementary Fig. 6

Supplementary Table 1

Supplementary Table 2

Supplementary Table 3

Supplementary Table 4

Supplementary Table 5

Supplementary Information
