# Supplementary figures and images for: DNA methylation, through DNMT1, has an essential role in the development of gastrointestinal smooth muscle cells and disease
Source: Cell Death Dis. 2018 Apr 27;9(5):474. doi: 10.1038/s41419-018-0495-z (PMC5920081; doi:10.1038/s41419-018-0495-z)

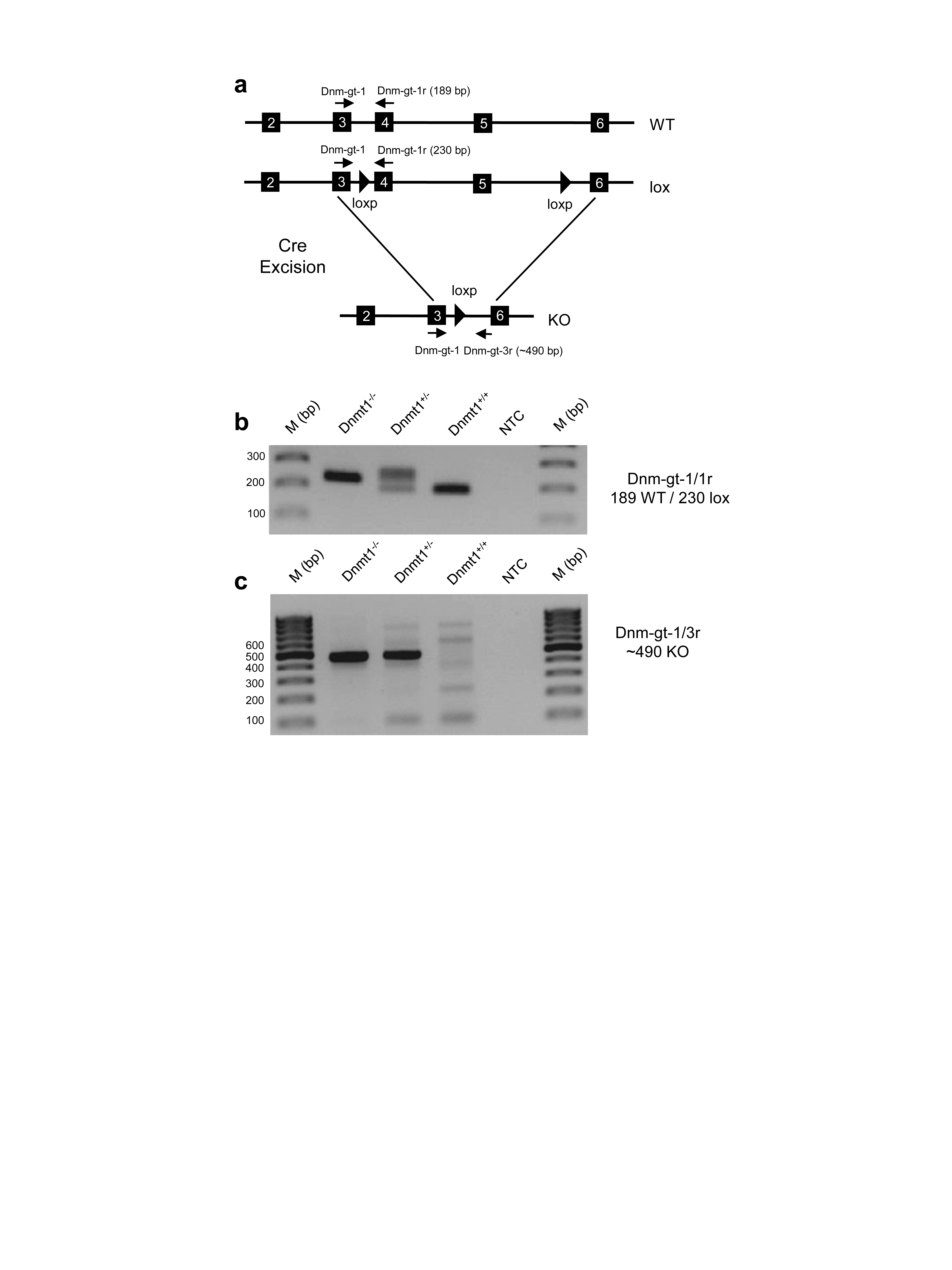

Supplement: Supplementary file 1 — Supplementary Fig. 1 [file 41419_2018_495_MOESM1_ESM.tif]

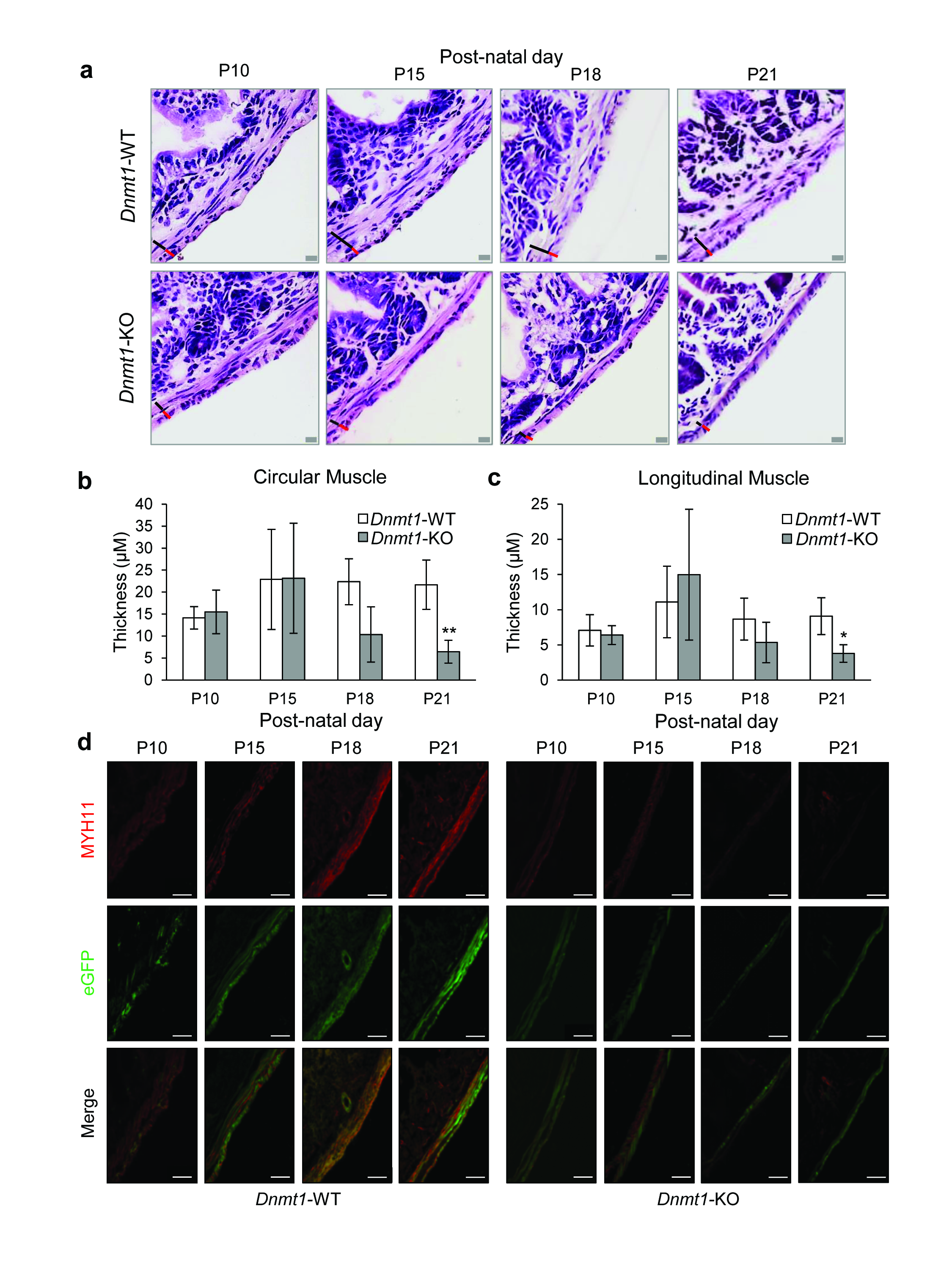

Supplement: Supplementary file 2 — Supplementary Fig. 2 [file 41419_2018_495_MOESM2_ESM.tif]

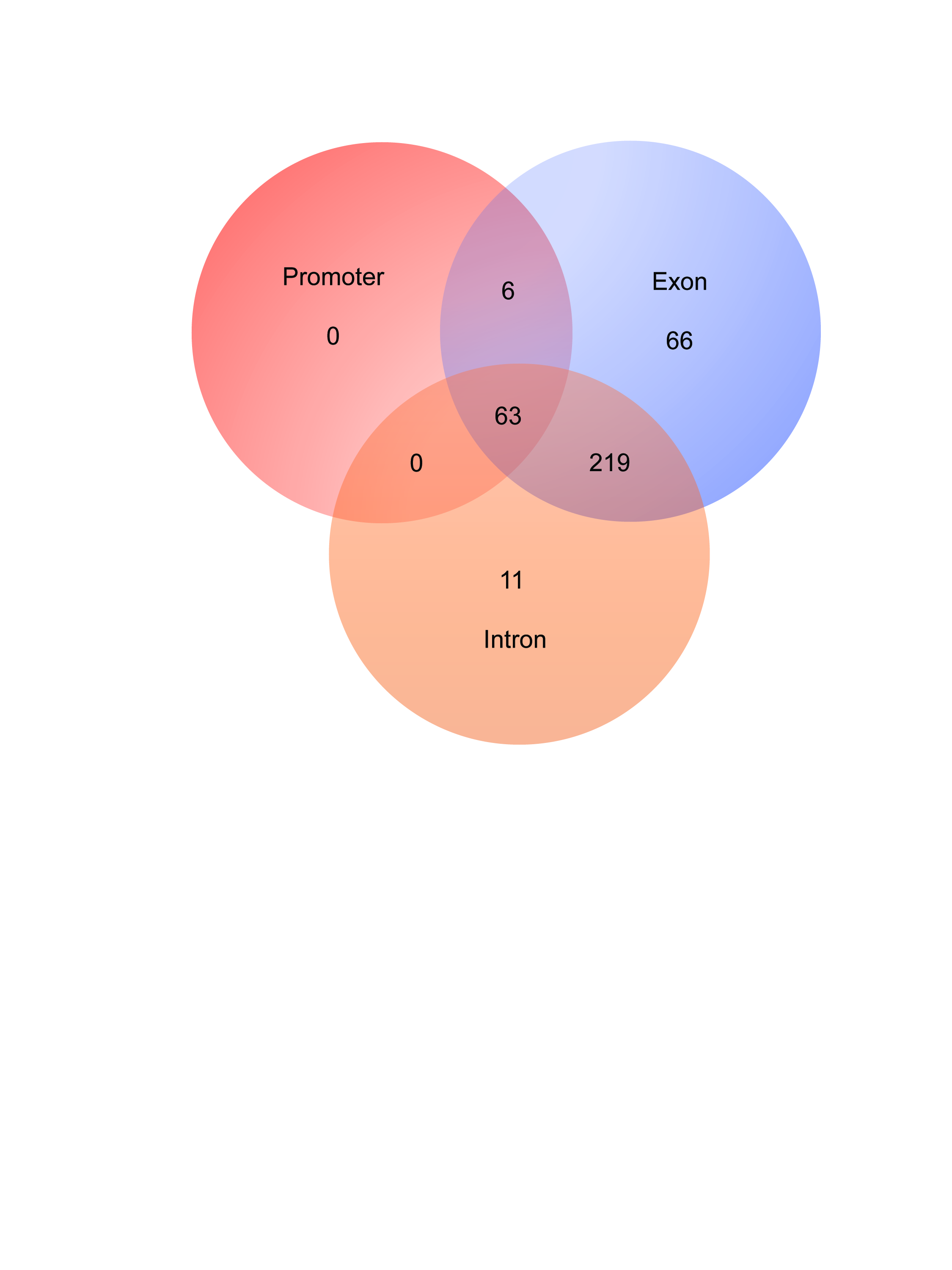

Supplement: Supplementary file 4 — Supplementary Fig. 4 [file 41419_2018_495_MOESM4_ESM.tif]

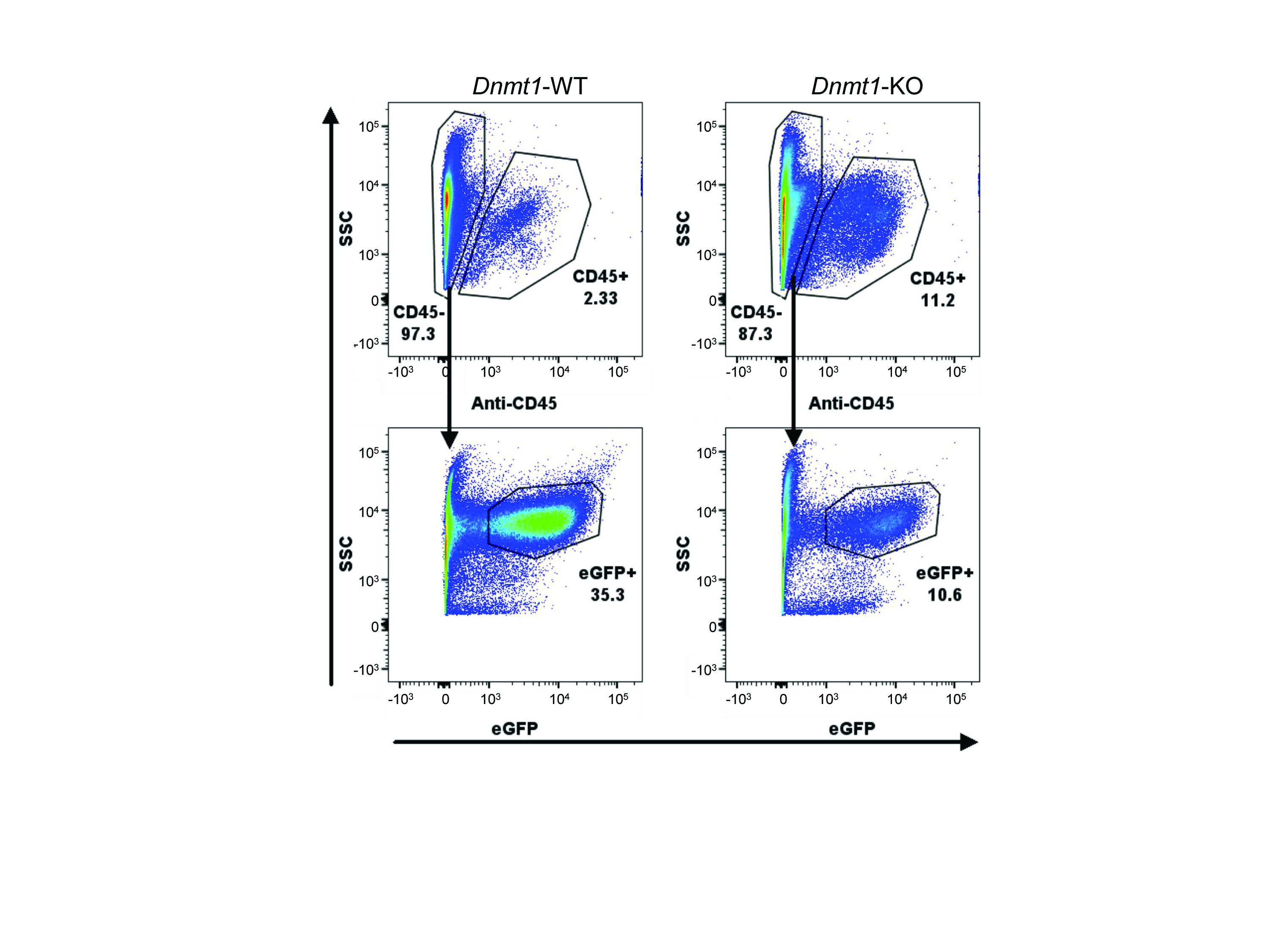

Supplement: Supplementary file 5 — Supplementary Fig. 5 [file 41419_2018_495_MOESM5_ESM.tif]

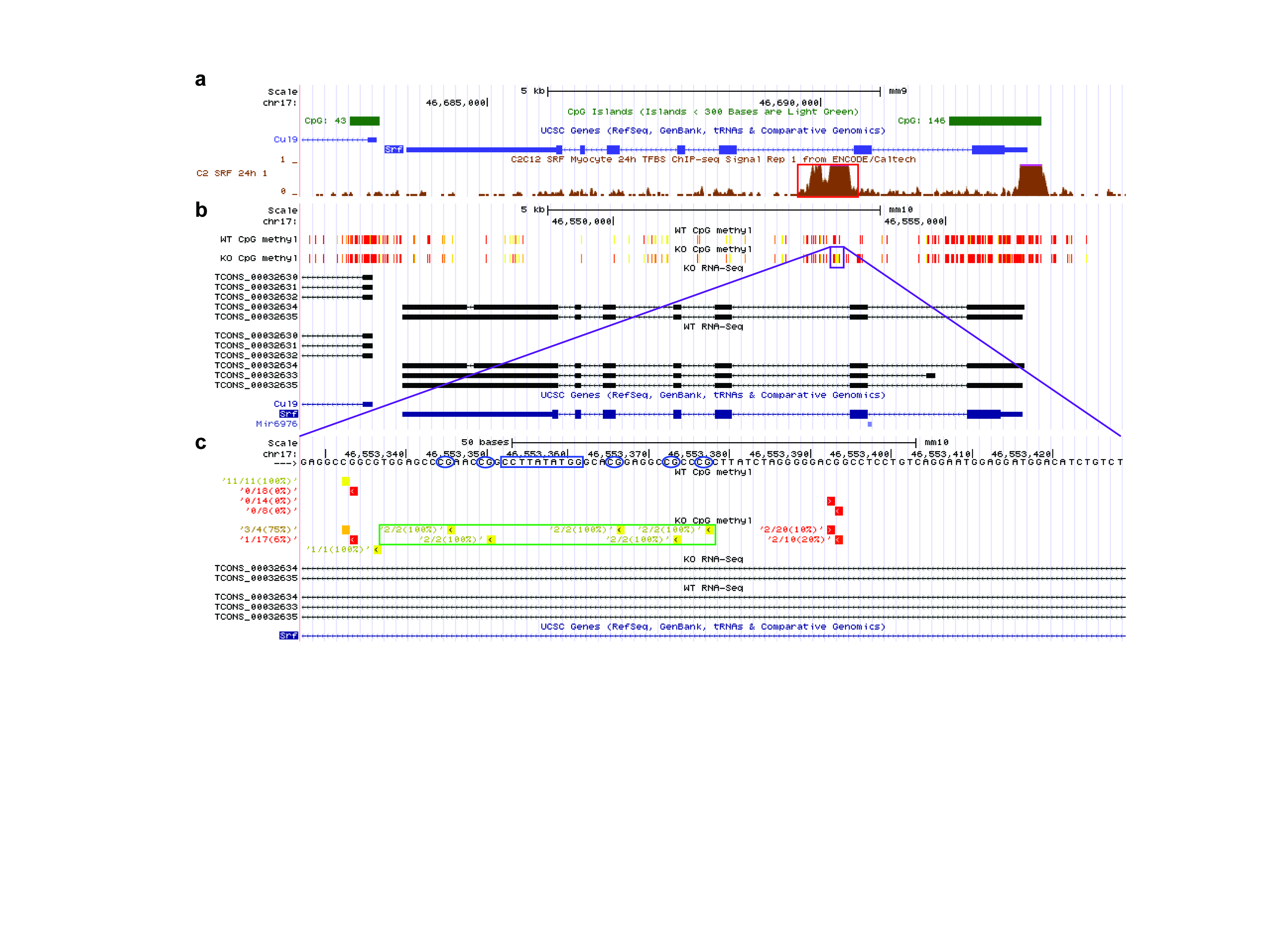

Supplement: Supplementary file 6 — Supplementary Fig. 6 [file 41419_2018_495_MOESM6_ESM.tif]
