# Supplementary material for: DNA methylation, through DNMT1, has an essential role in the development of gastrointestinal smooth muscle cells and disease
Source: Cell Death Dis. 2018 Apr 27;9(5):474. doi: 10.1038/s41419-018-0495-z (PMC5920081; doi:10.1038/s41419-018-0495-z)
Supplement: Supplementary file 7 — Supplementary Table 1 [file 41419_2018_495_MOESM7_ESM.docx]

**Supplementary Table 1.** Sequencing results of transcriptome (mRNA and miRNA) and DNA methylome from *Dnmt1*-WT and *Dnmt1*-KO tunica muscularis

***mRNA-Seq summary***

| Sample | **Total reads** | **Mapped reads** | **Annotated genes** |
| --- | --- | --- | --- |
| *Dnmt1*-WT | 341,290,850 | 291,592,121 | 16,632 |
| *Dnmt1*-KO | 252,046,246 | 206,053,958 | 17,055 |

***miRNA-Seq summary***

| **Sample** | **Total reads** | **Mapped reads** | **Annotated genes** |
| --- | --- | --- | --- |
| *Dnmt1*-WT | 11,100,926 | 5,725,664 | 956 |
| *Dnmt1*-KO | 10,221,072 | 4,768,158 | 979 |

***Methyl-MidiSeq summary***

| **Sample** | **Total Read (pairs)** | **Mapping** | **Unique CpGs** | **Average CpG coverage** | **Bisulfite conversion rate** |
| --- | --- | --- | --- | --- | --- |
| *Dnmt1*-WT | 240,783,359 | 53% | 15,259,699 | 9X | 99% |
| *Dnmt1*-KO | 237,493,706 | 53% | 14,680,291 | 11X | 99% |
