# Supplementary material for: DNA methylation, through DNMT1, has an essential role in the development of gastrointestinal smooth muscle cells and disease
Source: Cell Death Dis. 2018 Apr 27;9(5):474. doi: 10.1038/s41419-018-0495-z (PMC5920081; doi:10.1038/s41419-018-0495-z)
Supplement: Supplementary file 8 — Supplementary Table 2 [file 41419_2018_495_MOESM8_ESM.docx]

**Supplementary Table 2.** List of oligonucleotides and antibodies used in this study

***Oligonucleotides***

| **Genotyping Primers** | **Sequence (5’ to 3’)** | **Size (bp)** |
| --- | --- | --- |
| Dnm-gt-1 | GCCAGTTGTGTGACTTGGAAACC | 189 WT / 230 lox |
| Dnm-gt-1r | CTTGACTTTAGCCAGGTAGCC |  |
| Dnm-gt-1 | See above | ~490 KO |
| Dnm-gt-3r | GACTTGCAAAGTGGCAGGCAAG |  |
| Cre-2 | GCATGGTGCAAGTTGAATCGG | 140 |
| Cre-2r | GACCGACGATGAAGCATGTTTAGC |  |
| eGFP-2 | CTGACCCTGAAGTTCATCTGC | 130 |
| eGFP-2r | GACTTGAAGAAGTCGTGCTGC |  |
| **qPCR Primers** | **Sequence** | **Size (bp)** |
| Apoc1 Fwd | TCCTGATTGTGGTCGTAGCC | 80 |
| Apoc1 Rev | CTCCAATGTTCCGGACAAATCC |  |
| mCdkn1a-1 | GAACATCTCAGGGCCGAAAACG | 99 |
| mCdkn1a-1r | GGGCACTTCAGGGTTTTCTCTTG |  |
| Coro1a Fwd | CGCTCGCAGAAGAGCTACA | 82 |
| Coro1a Rev | ATTCCTCACGTCCTCCTCCA |  |
| Ctgf Fwd | AAGCTGACCTGGAGGAAAACA | 93 |
| Ctgf Rev | TGCAGCCAGAAAGCTCAAAC |  |
| Fcer1g Fwd | GTTCTTGCTCCTTTTGGTGGAA | 74 |
| Fcer1g Rev | GGACAGCATCCAGGATATAGCA |  |
| Fcgr3 Fwd | TCAGAATGCACACTCTGGAA | 82 |
| Fcgr3 Rev | ACTCTGCCTGTCTGCAAA |  |
| Gadd45g Fwd | AGAAGTCCGTGGCCAGGATA | 77 |
| Gadd45g Rev | GAAGTTCGTGCAGTGCTTTCC |  |
| Gapdh Fwd | CAAGGTCATCCCAGAGCTGAA | 82 |
| Gapdh Rev | CAGATCCACGACGGACACA |  |
| Gm1987 Fwd | ACACACACAGACCCCAACTT | 79 |
| Gm1987 Rev | GCCATGATTGTGGTTGAGTTGA |  |
| Krt18 Fwd | TCGAGGCACTCAAGGAAGAA | 79 |
| Krt18 Rev | GCAATCTGGGCTTCCAGAC |  |
| Myh11 Fwd | CCCAAGCAGCTAAAGGACAA | 76 |
| Myh11 Rev | AGGCACTTGCATTGTAGTCC |  |
| Nr4a1 Fwd | CAATGCTTCGTGTCAGCACTA | 104 |
| Nr4a1 Rev | TGTTTGCCAGGCAGATGTAC |  |
| Srf Fwd | ATCTGACAGCAGTGGGGAAA | 86 |
| Srf Rev | TCTGGATTGTGGAGGTGGTAC |  |
| Tnfrsf12a Fwd | TCGGGACCGGCAATCA | 80 |
| Tnfrsf12a Rev | ATCAACACCAAGCCGAATCC |  |
| Ubb Fwd | ATTCGGTCTGCATTCCCAGT | 65 |
| Ubb Rev | AATTGGGGCAAGTGGCTAGA |  |

***Antibodies***

| **Antibody^1^** | **Vendor (Item #)** | **Reactivity** | **Host** | **Clonality** | **MW^2^** | **Concentration** |
| --- | --- | --- | --- | --- | --- | --- |
| DNMT1  DNMT3a  DNMT3b  TET1  TET2  TET3  SRF  MYH11 (IHC)  MYH11 (WB)  SM22a  UBE1  Alexa^TM^ 594  Alexa^TM^ 488 | Santa cruz Biotech. (sc20701)  Santa cruz Biotech. (sc20703)  Abcam (ab16049)  Active motif (61443,611444)  Santa cruz Biotech. (sc136926)  GeneTex (GTX121453)  Santa cruz Biotech. (sc13029)  Santa cruz Biotech. (sc79079)  Abcam (ab124679  Abcam (ab124964)  Proteintech Group (15912-1-AP)  Jackson Immunoresearch (805-585-180)  Thermo-Fisher (A-11055) | Human, Mouse  Human, Mouse  Human, Mouse  Human, Mouse  Human, Mouse  Human, Mouse  Human, Mouse, Rat  Human, Mouse  Human, Mouse  Human, Mouse, Rat  Human, Mouse, Rat  Goat  Goat | Rabbit  Rabbit  Rabbit  Rabbit  Rabbit  Rabbit  Rabbit  Goat  Rabbit  Rabbit  Rabbit  Bovine  Donkey | Polyclonal  Polyclonal  Polyclonal  Polyclonal  Polyclonal  Polyclonal  Polyclonal  Polyclonal  Monoclonal  Polyclonal  Polyclonal  Polyclonal  Polyclonal | 184  101  95  223  212  194  51  NA  200  42  118  NA  NA | 1:100  1:100  1:100  1:100  1:100  1:100  1:500  1:200  1:500  1:2000  1:500  1:500  1:500 |

^1^IHC: Immunohistochemistry; WB: Western blot

^2^MW: Molecular weight (kDa)
