# Supplementary material for: DNA methylation, through DNMT1, has an essential role in the development of gastrointestinal smooth muscle cells and disease
Source: Cell Death Dis. 2018 Apr 27;9(5):474. doi: 10.1038/s41419-018-0495-z (PMC5920081; doi:10.1038/s41419-018-0495-z)
Supplement: Supplementary file 12 — Supplementary Information [file 41419_2018_495_MOESM12_ESM.docx]

**Supplementary Information**

**Supplementary Figure 1. PCR genotyping confirmation of *Dnmt1*-KO.** **a** Primers (Dnm-gt-1, Dnm-gt-1r) were designed to flank the inserted loxp sites between exon 3 and 4 (black boxes). A wild-type allele amplifies an amplicon of 189 bp while the inserted loxp site increases the amplicon length to 230 bp. To confirm excision of exons 4 & 5, primers (Dnm-gt-1, Dnm-gt-3r) were designed to amplify an amplicon of ~490 bp if knockout indeed occurred. **b**, **c** 2% agarose gel showing characteristic banding patterns for *Dnmt1*^-/-^, *Dnmt1*^+/-^ and *Dnmt1*^+/+^ for each previously listed primer set. The combination of a homozygous lox band (230 bp; Dnm-gt-1/1r), a knockout band (~490 bp; Dnm-gt-1/3r) and the presence of Cre (140 bp; Cre-2/2r, not pictured), as well as eGFP (130 bp; eGFP-2/2r, not pictured) bands was required for PCR confirmation of knockout.

**Supplementary Figure 2. Loss of smooth muscle layer in *Dnmt1*-KO.** **a** H+E images showing the loss of both the circular (black bars) and longitudinal (red bars) muscle layers in *Dnmt1*-KO mice. Scale bar (grey) is 10 µM. **b** The circular layer is the most drastically lost beginning at P18 yet it does not become a significant level of thinning until P21 (n=4, error bars are SD, ** = p < 0.01, unpaired t-test). **c** Similar to the circular layer, the longitudinal layer reaches significant levels of thinning at P21 (n=4, error bars are SD, * = p < 0.05, unpaired t-test). **d** Confocal images, in agreement with H+E images, showing the loss of the tunica muscularis through MYH11 staining in *Dnmt1*-KO mice beginning at P15 and confirms that *Dnmt1*-KO mice have no noticeable levels of MYH11 expression by P21. Scale bar (white) is 20 µM.

**Supplementary Figure 3. Expression profiles of smooth muscle markers and pro-apoptotic genes in the tunica muscularis of *Dnmt1*-WT and *Dnmt1*-KO mice**. *Dnmt1* shows significant loss at E18 and progressively loses expression over time in *Dnmt1*-KO mice while *Srf* and *Acta2* are not significantly lost until P15. Interestingly, *Myh11* begins to have a significant loss of expression at P9 that continues to wane through to P15 indicating that expression of *Myh11* can be regulated in a Srf-independent manner in *Dnmt1*-KO mice. Both pro-apoptotic genes found to be highly expressed in *Dnmt1*-KO isolated SMC, *Nr4a1* and *Gadd45g*, have at least a 4 fold increase in expression by P15 in *Dnmt1*-KO mice with *Nr4a1* showing significant increases in expression by P9. (n=3, error bars are SEM, * = p <0.05, ** = p <0.01, unpaired t-test)

**Supplementary Figure 4. A breakdown of the specific locations of significant hypomethylation within genes found to be upregulated in the tunica muscularis of *Dnmt1*-KO mice.** Overexpressed genes in *Dnmt1*-KO mice (392: FPKM > 10, >1 fold increase) were selected and analyzed for changes in CpG methylation. A majority of the upregulated genes found to have some level of hypomethylation (365/392), were found to have significant demethylation levels in both their exons and introns (219) with genes only having exons demethylated coming in next (66) followed by genes with hypomethylation in their promoters, introns, and exons (63), introns alone (11), and hypomethylation at only promoters and exons (6). There were no genes that only had their promoter demethylated or a combination of only promoter and intron demethylation.

**Supplementary Figure 5. *Dnmt1*-KO SMC lose eGFP and gain CD45^+^.** Of the cells isolated from the tunica muscularis through flow cytometry, 97.3% of cells in *Dnmt1*-WT are CD45^-^, and of those CD45^-^ cells, 35.3% of them had strong eGFP expression. These CD45^-^, eGFP^+^ cells represent isolated SMC. In cells isolated from the tunica muscularis of *Dnmt1*-KO mice, only 87.3% of all cells were CD45^-^ (approximately 5 fold increase in CD45^+^ cells) and of those CD45^-^ cells, only 10.6% of them had strong eGFP expression, indicating that the tunica muscularis is losing SMC and gaining cells of a hematopoietic lineage.

**Supplementary Figure 6. Intronic CArG box in *Srf* has surrounding aberrant CpG hypermethylation in *Dnmt1*-KO.** **a** ChIP-Seq results from C2C12 myocytes showing SRF binding sites, including the promoter region and a binding site in the second intron (red box). mm9 was used as reference genome as ChIP-Seq data for SRF is not available for mm10, and mm10 was used for all other sequencing/imaging. **b** This same *Srf* intronic region in *Dnmt1*-KO shows unexpected hypermethylation (purple box; yellow indicates higher levels of methylation, red indicates lower). *Dnmt1*-KO mice also show a complete loss of the *Srf* transcript, TCONS_00032633. *Srf* is transcribed from the antisense strand. **c** Expanded view of *Srf* intronic region. The CArG box found within this intronic region (sequence boxed in blue: CCATATAAGG in antisense strand) is flanked by hypermethylated CpG sites (blue ovals) in *Dnmt1*-KO (green box) that is not observed in *Dnmt1*-WT mice. This abnormal CpG hypermethylation could reduce access for transcription factors (including SRF itself), thus, reducing transcript levels of *Srf*, possibly contributing to the elimination of the transcription of TCONS_00032633 in *Dnmt1*-KO mice resulting in the exaggerated phenotypic symptoms seen from P15-P21.

**Supplementary Table 1.** Sequencing results of transcriptome (mRNA and miRNA) and DNA methylome from *Dnmt1*-WT and *Dnmt1*-KO tunica muscularis

**Supplementary Table 2.** List of oligonucleotides and antibodies used in this study

**Supplementary Table 3.** Expression profiles of mRNA-seq (**a**) and miRNA-seq (**b**) between *Dnmt1-WT* and *Dnmt1-KO* tunica muscularis

**Supplementary Table 4.** List of gene ontology (GO) terms derived from functional categories associated with genes upregulated in *Dnmt1-KO* tunica muscularis

**Supplementary Table 5.** Analysis of genomic CpG methylation in promoter (**a**), exonic (**b**), and intronic regions (**c**) between *Dnmt1-WT* and *Dnmt1-KO* tunica muscularis
